# Self-assessments, attitudes, and motivational orientations towards the use of digital media in teaching a comparison between student teachers of different subject clusters

**DOI:** 10.1016/j.heliyon.2023.e19516

**Published:** 2023-08-29

**Authors:** Marcus Brändle, Christina Sotiriadou, Bernd Zinn

**Affiliations:** University of Stuttgart, Institute of Educational Science (IfE), Department of Vocational Education Focused on Teaching Technology (BPT) Germany

**Keywords:** TPACK, Digitalisation, Teaching, Attitudes, Motivational orientation, Digitalisation-related competencies, Student teachers

## Abstract

Findings from research on the education of teachers show that student teachers lack competence in the utilization of digital media and often fail to see the added value in using digital concepts in classrooms. Teacher training institutions are faced with the central challenge of providing student teachers and teachers with adaptive learning opportunities for their competence in digital areas. In the context of various teaching subjects, this raises questions about specific starting points and the actual needs of students, as well as university course offerings. The present study tackles this topic and explores the commonalities and differences between student teachers from three subject clusters: science, technology, engineering, and mathematics (STEM); language literature arts (LLA); and social sciences (SOCS). The questionnaire-based study examines student teachers' self-assessments of their abilities in terms of digital media, attitudes, and motivational orientations towards the use of digital media in teaching. Group comparisons show that STEM student teachers rate themselves better in competence facets such as technological knowledge, technological content knowledge, and technological pedagogical knowledge, although their attitudes and motivation towards learning with digital media do not differ. Despite the different interrelation effects of investigated influencing factors on motivation depending on subject clusters, the findings suggest the promotion of technological competencies and positive attitudes to increase motivation.

## 1 Introduction

Teacher training universities, as well as the second and third teacher training phases, have a significant role to play within the context of digital transformation. Ongoing digitisation is changing education in terms of the design of teaching and the process of learning upon which teaching is based. Teacher training institutions have a special responsibility to enable (prospective) teachers to acquire or expand their digitisation-related competencies within the framework of professionalisation so that they can use new digital technologies and corresponding concepts for teaching and learning development. According to the findings of research on professionalisation, competent teachers ensure that children and young people in school are optimally prepared for their future life and work, and preparation is constantly changing because of digitalisation [e.g. 1]. Teachers must be able to leverage modern digital technologies to facilitate and improve the achievement of subject-specific and interdisciplinary educational goals. The use of digital technologies in the classroom, such as virtual reality or explanatory videos, is not an end but must be linked to the expectation of added value for teaching and learning. However, where does added value come into play?

A meta-analysis by Hillmayr et al. [2] reports that the use of digital technology in teaching mathematics and science results in positive effects on learning performance, with moderate to strong effect sizes based on 92 studies. Even if the use of digital technologies is promising based on existing studies, the state of research shows clear disparities in terms of the effect on the subject matter. While greater effects have been reported in the context of natural science subjects, negligible effect were shown for social science subjects, and language and mathematics subjects were in the middle [[2], [3], [4], [5]].

This raises the question of the causes of subject-specific differences. There can be many reasons for this, whereby one causality for lesser effects can be based on the digitisation-related competencies of teachers themselves. Studies on student teachers show that their digitalisation-related competencies require development [6], hardly experience any progression in the course of their studies [e. g. 7], and they have little digital affinity [8]. Compared to other student groups, student teachers use digital media the least and, according to the findings of Schmid and colleagues, show the least motivation for using digital media [6]. To find possible starting points for the implementation of digitalisation training in university teaching, we analyzed the motivation of student teachers in using digital media in the classroom. In this context, we are interested in the extent to which there are different perspectives on competency in digitalisation between different teacher training subjects and which factors analytically explain the motivation to use digital media in the classroom.

## 2 Theoretical background and state of research

In this chapter, after presenting the theoretical background of attitudes and motivational orientation (Section 2.1), the assumptions of the modelling of teachers' competencies in digitisation relevant to our own study are described in Section 2.2. Subsequently, the state of research considered relevant to the study is presented in Section 2.3.

### 2.1 Attitudes and motivational orientation towards using digital media in learning

According to Weinert [9], the professional competence of prospective teachers includes not only professional knowledge but also the associated attitudes and motivational orientations [also 10]. Particularly in teaching practice, positive attitudes and the corresponding motivational orientations are of great importance for successfully interpreting and coping with challenging teaching situations [11,12]. The pedagogically and didactically meaningful integration of digital media in everyday school life is thus not only determined by digitisation-related knowledge and the skills of a teacher but also by their attitude towards using digital media for learning [e.g., [13], [14], [15]] and their motivation to use digital media in the classroom.

[10,16]. Although there are different definitional approaches to the psychological construct of attitudes, the research literature largely agrees that attitudes are defined as "evaluations of objects, people, groups and other kinds of objects in our social world" [17 p. 198], which are based on cognitive, affective, and behavioural information processes [18]. Ajzen [19] further argues that attitudes include positive and negative judgements that arise from our beliefs and individual experiences and are key indicators of a person's intention to perform a particular behaviour. The literature states that actions are significantly guided by attitudes [20,21] and that behaviour and experiences influence attitudes [22].

In a bipolar representation, the attitudes of teachers in the reference field of digitalisation-related competencies represent the expression of a favourable or unfavourable pedagogical attitude towards the use of digital media in the classroom for student learning [23]. The use of digital media in the classroom can, for example, be associated with positive attitudes concerning more efficient learning processes, improved learning outcomes, and motivational effects. On the other hand, fears and negative evaluations of media use in the classroom can also determine attitudes [24,25].

Attitudes are contextual constructs, meaning that attitudes towards digital media can vary in different contexts (e.g., inclusion and digitisation) [26,27]. For example, teachers may report the frequent private use of digital media and be open to its use in principle, but at the same time, they are not convinced of the benefits and potential uses of digital media in teaching practices [[28], [29], [30]]. Eagly and Chaiken [18]; therefore, they refer to attitudes as the "psychological tendency to evaluate", since attitudes and the associated judgements are constantly constructed depending on current contextual information and experiences [31,32]. New (individual and positive) learning experiences in technology courses [33] or other technology-supported learning environments [34] can sustainably improve teachers' attitudes towards the use of digital media in the classroom. Studies on the acceptance of technology also suggest that attitudes influence teachers' perceptions and behaviour and thus their acceptance and motivation to integrate digital media into the classroom or to engage with it. Motivation orientation is an important component of teaching professionalism in the context of technological education, as it is understood as a "process whereby goal-directed activity is instigated and sustained" [35 p. 4]. Based on the theory of reasoned action [20, TRA, 36], the theory of planned behaviour [TPB, 37, 38], and the technology acceptance model [TAM, 39], it can be assumed that the attitude towards digital media in the classroom determines the intention, i.e., the behavioural intention to use digital technologies in the classroom. In turn, the resulting motivation influences the actual behaviour in the classroom [20,22]. Zhang et al. [40] suggest that students' behavioural attitudes towards the use of digital technologies are a significantly stronger predictor of behavioural intention than object-related attitudes in the sense of a generally positive or negative evaluation of an object [40]. They are of the opinion that the attitude towards certain behaviours, such as the use of digital media in class, must be specifically promoted and not only the attitude towards digital media per se in order to sustainably improve the intention to use digital media.

### 2.2 Digitalisation-related competencies of teachers

There are numerous concepts and models for the description of digitisation-related competencies of teachers with conceptual diversity and different claims of meaning [27, overview, e.g., 41]. For this article, we use the term "digitisation-related competences" in the sense of van Ackeren et al. [1, p. 108].^1^

The diversity of the content of the different concepts and models reflects a rapidly changing and increasingly digitalised media world, as well as the reference to various subject disciplines [[42], [43], [44]]. Relevant international and national digitisation-related competence models include the Will–Skill–Tool and Pedagogy Model [45], the European Framework for the Digital Competence of Educators Model [DigCompEdu; 46], and the integrative model of digitisation-related competencies for teacher training from the University of Duisburg-Essen [UDE Model; 47].

An internationally significant and prominent model is the technological pedagogical content knowledge model [TPACK; 48, 49]. The TPACK model refers to the complexity of teaching processes and describes the structure of the dynamic interaction of subject-specific and non-subject-specific knowledge areas, which should enable teachers to use technologies efficiently when teaching [49]. The starting point of this model is the theoretical concept postulated by Ref. [50], which defines pedagogical knowledge (PK), content knowledge (CK), and pedagogical content knowledge (PCK) as the core knowledge areas of teachers' professional competence. PCK encompasses knowledge about teaching and learning processes, while CK includes content knowledge about the concepts, theories, and structures of a specific subject. The overlap of these two areas of knowledge (i.e., PCK) describes the knowledge required for the appropriate presentation and communication of subject content. Against the background of the increasing digitalisation of teaching and learning processes, Mishra and Koehler [49] extended Shulman's conception to include a technology-related knowledge component, which includes knowledge about the application of various (educational) technologies and itself has interfaces with the other knowledge areas. The model for describing digitisation-related competencies encompasses both knowledge about the way in which digital media and technologies can be used to present and convey subject-related content (technological content knowledge, TCK) and knowledge about the possible pedagogical and didactic potential of various digital media and technologies to support teaching and learning processes (technological pedagogical knowledge, TPK). Finally, the central determinant of successfully using digital media for teaching is the intersection of all three areas of knowledge: technological pedagogical content knowledge (TPACK). TPACK thus combines the knowledge for a pedagogically and didactically meaningful use of digital technologies in subject- or content-specific teaching [49]. Internationally, the TPACK model is one of the best-studied empirical models of professional teacher knowledge [[51], [52], [53], [54], [55]] and enables good comparability with a large number of empirical and practice-oriented research projects in the field of digitisation-related competencies in teacher education [56]. In addition, the TPACK model is based on the standard models of educational research [10,50] so it appears to be particularly compatible both theoretically and in terms of educational practice [7, e.g., 54]. Due to its proven use in the development of offers for training the digitisation-related competencies of teachers [[57], [58], [59]], the model appears to be useable for the present study as a basis for analysing the self-assessments of the digitisation-related competencies of student teachers.

### 2.3 State of research

As mentioned in the Introduction, beginning and advanced teacher students display deficits in digitisation-related competencies compared to other students. This is particularly true for students that are not in STEM subjects [6]. Studies on student teachers show that they tend to rate their digitalisation-related competencies low. In addition, the findings indicate that these self-assessments do not develop significantly during the course of studies [7]. According to Schmid et al. [28], low digitisation-related competencies can lead to lower motivation in the use of digital media overall and a lower use of digital media in university teaching–learning contexts. Studies suggest that the digitisation-related competencies of (prospective) teachers significantly influence the decisions and behaviour that impact the use of technology in the classroom [[60], [61], [62]].

This enforces the poorly perceived didactic usefulness of digital teaching–learning scenarios in terms of improving their own teaching quality or the impact on students' learning outcomes [28,63]. The findings thus imply that, in addition to digitisation-related knowledge and skills, the individual attitudes of student teachers towards the integration of digital media in teaching contexts must also be considered as a decisive influencing factor [64]. This connection has already been demonstrated several times [23, e.g., 65, 66]. Guggemos and Seufert [65] differentiate between the use of technologies and the teaching of technology-related content in the classroom. In their analysis of Swiss vocational school teachers in the classroom, they present a structural equation model for predicting these two possible uses, which considers various technology-related TPACK and attitudinal facets. The results of their analysis indicate that the use of technology in the classroom is directly and positively predicted by the subject-independent competence facet TPACK and by attitudes towards technology in the classroom with a variance explanation of 36%. In contrast, TPACK and the attitude towards technology-related content in the classroom were significant for the teaching of technology-related content in the classroom. In their analysis, TK also shows an indirect influence on the use of technologies and technology-related content in the classroom via TPK. The findings of this study led to the conclusion that in the education and training of teachers, the sole promotion of TK is not sufficient, and TPK and TPACK as well as attitudes towards technologies and technological knowledge merited focus (ibid.). Quast et al. [66] also confirmed that the teachers' use of digitally supported teaching strategies is determined by individual values (importance, interest, and usefulness) and digital competence self-assessments. From this, it can be concluded that teachers must perceive themselves as digitally competent and that they must recognise the value of using digital media in order to use digital media for implementing quality teaching processes. There are broadly comparable empirical findings between teachers and student teachers in the field of reference [23, e.g., 65, e.g., [67], [68], [69]]. In their study, Vogelsang et al. [23] surveyed student teachers with at least one science subject. Their findings show that student learning experiences can significantly and positively influence attitudes and self-efficacy expectations towards media use in the classroom, which can positively predict motivational orientation towards digital media use. In their explanatory model for motivational orientation, 43.1% of the variance was explained, with attitudes towards media use having the greatest influence. Scherer et al. [64] take an extended look at the relationship between TPACK and attitudes towards using digital media for learning, which have been considered independent predictors of motivational orientation in existing studies. Their results show that better attitudes towards the use of digital media for learning condition better assessments in both the pedagogical facets of TPACK (including TPK, TCK, and TPACK) and TK. The findings on the correlations between attitudes and TPACK are heterogeneous overall [23, e.g., 65]. In addition to the influencing factors described, further study findings indicate that the contextual framework conditions of the subject as well as the available technical equipment can exert an influence on teachers’ motivation in addition to the stable and empirically more easily accessible facets of attitudes and TPACK [70].

To summarise the state of research, several studies on the influence of the attitude or the competence assessment of TPACK facets on the motivation to use digital media by (prospective) teachers are available on a cross-subject level. The empirical findings provide fragmented information on the following subject clusters: STEM (science, technology, engineering, and mathematics), LLA (language, literature, and arts), and SOCS (social sciences). According to the data available to us, there is a lack of a systematic description of digitisation-related competencies between different subject clusters in the teacher training programme, which would make it possible to provide both subject-specific and cross-subject descriptive knowledge within an investigation. In addition, an analysis of factors influencing the motivational orientation towards the use of digital media in the classroom with a differentiated consideration of subject-specific contexts (in mathematical–scientific, linguistic–literary–artistic, and socio-scientific teacher training areas) does not seem to have been sufficiently clarified so far. Based on systematically collected data, well-founded indications could be derived in each reference area for the content design of subject-specific and/or interdisciplinary university teaching events.

## 3 Study design

The present study addresses the research desiderata described above and aims to provide information on the subject-specific assessments, attitudes, and motivations of STEM, LLA, and SOCS student teachers in the context of their digitalisation-related competencies (research objective 1). Furthermore, the study aims to show the influences of self-assessments on the efficacy and attitudes of student teachers towards using digital media for learning and their motivational orientation towards their use of digital media in teaching (research objective 2).

### 3.1 Research questions and hypotheses

Based on the objective, the following two research questions (RQ 1 and RQ 2) and the following hypotheses are formulated based on the state of research reported in the second section.

Research question 1: How do student teachers assess their digitalisation-related competencies in a comparative, subject-cluster-specific view in terms of the seven knowledge facets of the TPACK model (TK, CK, PK, PCK, TCK, TPK, and TPACK), the attitudes towards using digital media for learning (ALDM), and their motivational orientation with respect to the use of digital media in teaching (MUDM)?

Hypotheses 1 to 4: The student teachers in the STEM cluster will rate themselves as significantly more competent in each of the technology-related facets of the TPACK model (H1 to TK, H2 to TPK, H3 to TCK, and H4 to TPACK) compared to the student teachers in the LLA and SOCS clusters.

Hypothesis 5 Student teachers in the STEM cluster have significantly more positive attitudes towards using digital media for learning (ALDM) than student teachers in the LLA and SOCS clusters (H5).

Hypothesis 6 The motivational orientation towards the use of digital media in teaching (MUDM) is significantly higher among student teachers in the STEM cluster compared to student teachers in the LLA and SOCS clusters (H6).

Research question 2: Do the assessments in terms of each of the technology-related facets of the TPACK model (TK, TPK, TCK, and TPACK) as well as the attitudes towards using digital media for learning (ALDM) influence the students' motivational orientation for the use of digital media in the classroom (MUDM)?

### 3.2 Description of survey instruments

TPACK knowledge facets (TK, CK, PK, PCK, TCK, TPK, and TPACK) were assessed using a translated questionnaire from Schmidt et al. [71] and its extended form provided by Zinn et al. [7]. The constructs on attitudes towards using digital media for learning (ALDM) and motivational orientation towards the use of digital media in the classroom (MUDM) were assessed using the scales adapted from Vogelsang et al. [23]. A five-point Likert scale was used for the seven TPACK constructs, and the items on attitude and motivational orientation are based on a four-point Likert scale. Table 1 provides an overview of the total constructs surveyed with an exemplary item from each of the scales used. Test subjects were recruited via mailing lists at universities. The questionnaire was completed online by the surveyed students. Participation in the survey was voluntary and can only be started with the consent of the students. Data were recorded pseudonymously. The ethics committee of the University of Stuttgart the reviewed ethical and legal aspects of the study and approved the study's execution.

**Table 1:** Scale analysis and sample items of the recorded constructs [7 (translated), 23 (translated), 71].

Scale	Number of items in the questionnaire	Reliability of the statement	Subject-specific	Example item
TK	6	0.88	X	I have the technical skills I need to use technology.
CK	3	0.81	✓	I have sufficient subject knowledge in my 1st subject.
PK	6	0.84	X	I can adapt my teaching style to different learners.
PCK	6	0.84	✓	I can help my students understand the knowledge of my 1st subject in a variety of ways.
TCK	5	0.84	✓	I know about digital technologies that I can use to help students better understand and apply the content of my 1st subject.
TPK	9	0.91	X	I can use strategies that combine content, technologies, and teaching approaches that I learned about in my coursework in my classroom.
TPACK	8	0.91	✓	I can combine subject knowledge of my 1st subject, digital technologies, and teaching methods in the classroom in such a way that they support the teaching process efficiently.
ALDM	8	0.85	X	Digital media should generally be given a strong weighting in school curricula.
MUDM	6	0.88	X	I am very excited to consider how I can better support the learning of my (future) students using digital media.

### 3.3 Sample and data analysis

After excluding incorrect or duplicate questionnaires, the sample included a total of n = 759 (f = 72.6%, m = 27.1%, d = 0.3%) students from all teacher training courses (L1 to L5) at five universities in Baden-Württemberg and Bavaria. Data collection took place each semester over four survey waves, ranging from the summer semester of 2020 to the winter semester of 2021/22. The motivation to use digital media in the classroom and the attitudes towards using digital media for learning were only recorded in the last survey wave for *n*_*MUDM/ALDM*_ = 162 students (f = 74.1%, m = 25.9%). The distribution of the sample was based on the studied teaching subjects. In total, the respondents studied 567 STEM subjects, 361 social science subjects, and 437 linguistic/literary/artistic subjects. People who studied two subjects in the same subject cluster were only included once in the analysis. This decision avoids duplications in the allocation and resulting distortion of descriptive statistics.

Data analyses were conducted using the statistical software R [72]. All calculations are based on an alpha level of 0.05. There was no normal distribution of the raw data (Shapiro–Wilk for all scales p < 0.05). No outlier values were excluded from the raw data. Using stepwise linear, a model with the highest possible variance explanation for the motivational orientation of the use of digital media in the classroom was determined, including all TPACK facets as well as the attitude towards using digital media for learning. The level for variance inflation factors (VIFs) is set at VIF <5 [73]. Additionally, a non-parametric model check using 10,000 BCa bootstrap samples was conducted to underpin the model's stability due to small sample sizes. To look at the interrelationships of variables within regression models with the highest variance resolution, mediation analyses were conducted using PROCESS [74]. For reasons of comprehensiveness, only the regression model of the respective observation providing the highest variance explanation was reported.

## 4 Results

### 4.1 Examination of the self-assessment by subject cluster (RQ 1)

**Fig. 1:**
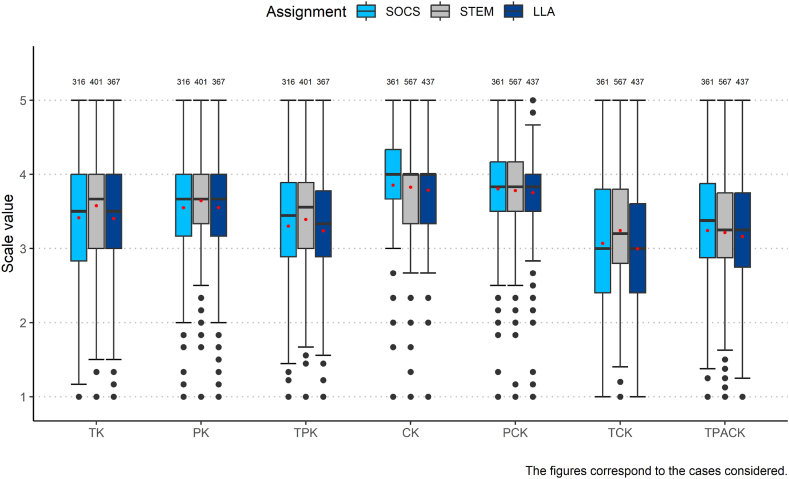
Box and whisker plot showing median (horizontal bars) and mean values (red dots) of the TPACK knowledge areas depending on the subject cluster (SOCS = social sciences; STEM = science, technology, engineering, and mathematics; LLA = language, literature, and arts).

In this section, the assessments of the seven areas of knowledge surveyed are subjected to an analysis of variance according to subject clusters. The box and whisker plots in Fig. 1 provide an overview of the assessment within the SOCS, STEM, and LLA clusters.

Table 2 shows the statistical parameters of the comparative ANOVAs of the subject clusters (Fig. 1). The Tukey–Kramer post hoc tests show significant group differences with lesser effect sizes between the STEM and SOCS groups and a highly significant group difference between STEM and LLA regarding technological content knowledge (TCK; *d*_*STEM-LLA*_ = 0.29; *d*_*STEM-‍SOCS*_ = 0.19). In the assessment of technological knowledge (TK; *d*_*STEM-LLA*_ = 0.23; *d*_*STEM-‍SOCS*_ = 0.20), the LLA and STEM groups differ extremely significantly, and SOCS and STEM differ significantly. With respect to the technological pedagogical knowledge (TPK; *d*_*STEM-LLA*_ = 0.21) scale, significant differences between the STEM and LLA subject clusters were also confirmed but not between STEM and SOCS.

**Table 2:** ANOVAs on differences between STEM, LLA, and SOCS subject clusters.

	Mean STEM (Med, SD)	Mean LLA (Med, SD)	Mean SOCS (Med, SD)	ANOVA	Tukey-HSD post hoc
CK	3.83 (4.00, 0.72)	3.78 (4.00, 0.70)	3.85 (4.00, 0.76)	*F*(2, 1362) = 0.88	STEM-LLA STEM-SOCS SOCS-LLA
PCK	3.78 (3.83, 0.60)	3.75 (3.83, 0.59)	3.80 (3.92, 0.68)	*F*(2, 1362) = 0.67	STEM-LLA STEM-SOCS SOCS-LLA
PK	3.64 (3.67, 0.64)	3.55(3.67, 0.70)	3.55 (3.67, 0.73)	*F*(2, 1081) = 2.26	STEM-LLA STEM-SOCS SOCS-LLA
TK	3.57 (3.67, 0.77)	3.40 (3.50, 0.74)	3.41 (3.50, 0.82)	*F*(2, 1081) = 5.95**	STEM-LLA** STEM-SOCS* SOCS-LLA
TPK	3.40 (3.56, 0.72)	3.24 (3.33, 0.78)	3.31 (3.44, 0.78)	*F*(2, 1081) = 3.81*	STEM-LLA* STEM-SOCS SOCS-LLA
TCK	3.24 (3.20,0.87)	2.99 (3.00, 0.85)	3.08 (3.10, 0.91)	*F*(2, 1362) = 10.35***	STEM-LLA*** STEM-SOCS* SOCS-LLA
TPACK	3.22 (3.25, 0.77)	3.15 (3.25, 0.79)	3.25 (3.38, 0.83)	*F*(2, 1397) = 1.04	STEM-LLA STEM-SOCS SOCS-LLA
ALDM	3.12 (3.12, 0.52)	3.05 (3.00, 0.51)	3.02 (3.12, 0.60)	*F*(2, 234) = 0.76	STEM-LLA STEM-SOCS SOCS-LLA
MUDM	2.79 (2.83, 0.71)	2.72 (2.83, 0.71)	2.68 (2.67, 0.68)	*F*(2, 234) = 0.49	STEM-LLA STEM-SOCS SOCS-LLA

Calculation if variance homogeneity is satisfied: ANOVA. Significance: *** <0.001, ** <0.01, and * <0.05.

The analysis of group differences between the STEM and LLA or SOCS subject clusters regarding attitudes towards using digital media for learning (ALDM) (H5) and motivational orientation in the use of digital media in the classroom (MUDM) (H6) did not reveal any significance. As expected, there are no statistically significant differences between subgroups STEM, LLA, and SOCS in the self-assessments for knowledge areas CK, PCK, and PK.

Thus, hypotheses H1 (TK) and H3 (TCK), both in comparison with the LLA group and with the SOCS group, and H2 (TPK) with the LLA subgroup are confirmed. Hypothesis H4 (TPACK) is rejected. Hypotheses H5 and H6 are also rejected in group comparisons. This means that the investigated attitudes towards using digital media for learning (ALDM) and the motivational orientation towards the use of digital media in the classroom (MUDM) are independent of the subject clusters for the considered sample. Due to the heterogeneous findings of the hypothesis testing, various factors influencing the motivational orientation towards using digital media for learning will be examined in the following, both across subjects and their subject clusters.

### 4.2 Investigation of the influences on the motivational expression for using digital media to learn (RQ 2)

In a stepwise regression, we determined which independent variables observed in the study (ALDM and assessments of the TPACK knowledge facets: *TK, CK, PK, PCK, TCK, TPK,* and *TPACK*) predict the motivational orientation towards the use of digital media (dependent variable: MUDM) with the best possible variance explanation. First, a cross-disciplinary analysis of the correlations was carried out.

**Fig. 2:**
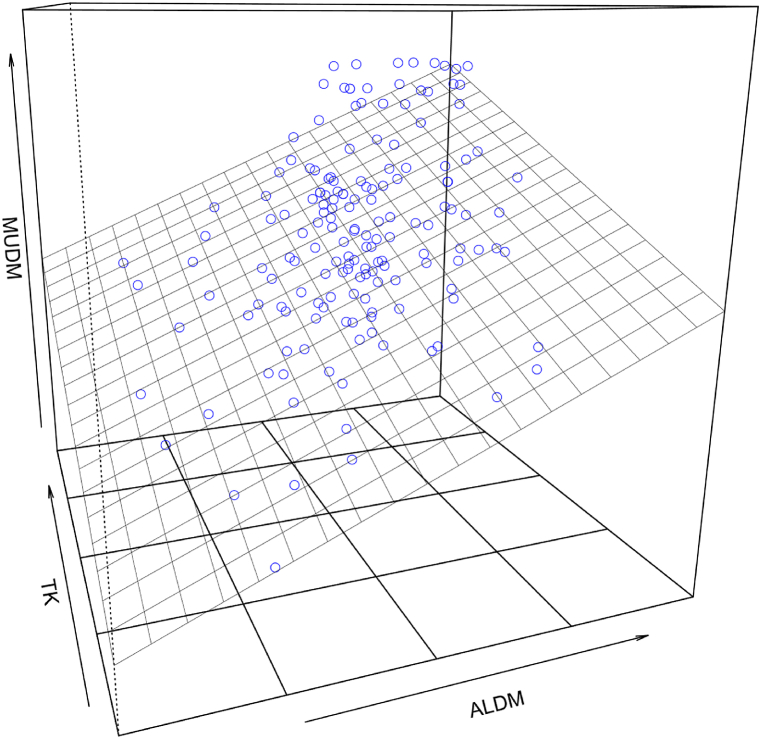
Visualization of the 3D regression model of TK and ALDM in terms of the motivational orientation towards the use of digital media in the classroom (MUDM).

The final regression model with the three variables for predicting attitudes towards using digital media for learning (ALDM), the assessment of technological knowledge (TK), and technological pedagogical knowledge (TPK) yielded a variance explanation of *R*^*2*^ = 0.45 (*β*_*ALDM*_ = 0.39, *t*_*ALDM*_ = 7.55, *p*_*ALDM*_ < 0.001, 95% CI_ALDM_ [0.265, 0.484]; *β*_*TK*_ = 0.30, *t*_*TK*_ = 5.65, *p*_*TK*_ < 0.001, 95% CI_TK_ [0.199, 0.397]; *β*_*TPK*_ = 0.17, *t*_*TPK*_ = 3.17, *p*_*TPK*_ < 0.01, 95% CI_TPK_ [0.065, 0.299]; *F(3, 231)* = 62.51; *p* < 0.001).^2^ This model is characterized by the highest variance resolution for the scales observed across subjects, with a concurrent satisfactory value for VIF <5 (*VIF*_*ALDM*_ = 1.09, *VIF*_*TK*_ = 1.40, and *VIF*_*TPK*_ = 1.36). Fig. 2 shows an example of the 3D regression model with the two strongest predictors (TK and ALDM) on dependent variable MUDM.

**Fig. 3:**
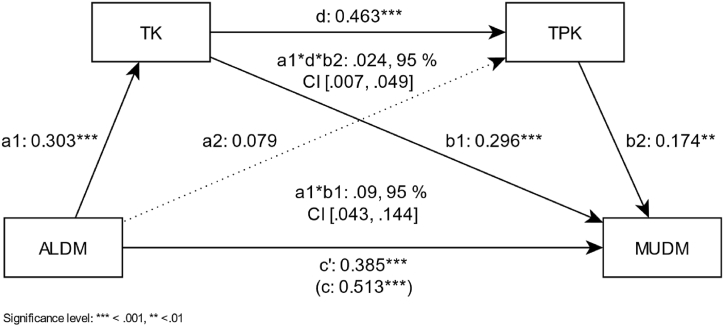
Mediation model with the standardized regression coefficients of the cross-curricular analysis of the factors influencing the motivation to use digital media in the classroom.

To further investigate the effect relationships of the individual predictors of the model with the highest variance explanation on motivational orientation, a serial multiple mediation analysis was then conducted. The results of this multidisciplinary mediation analysis (Fig. 3) show a significant positive effect of attitude (ALDM) on the motivation to use digital media in the classroom (total effect, *β* = 0.513, *p <* 0.001, *R*^*2*^_*ALDM-MUDM*_ = 0.263).

With the inclusion of mediators in the model, attitudes towards using digital media for learning (*β* = 0.385, *p* < 0.001), *TK* (*β* = 0.296, *p* < 0.001), and *TPK* (*β* = 0.174, *p <* 0.001) are shown to be significantly positive predictors of motivation. Thus, the direct path of the effect of attitude on motivation is weaker after including mediators in the model but remains significant. The 95% bootstrap confidence interval is also statistically significant (a1*d*b2 = 0.024, 95% CI [0.007, 0.049]) for the standardized indirect effect. The effect strength of this partial mediation (path: a1*d*b2) is weak. Nevertheless, this effect suggests that the relationship between attitude and motivation is mediated by TK and TPK, in which a higher value of attitude predicts a higher estimation in TK (*β* = 0.303, *p* < 0.001). This, in turn, leads to a higher estimation in TPK (*β* = 0.463, *p* < 0.001) and, as a result, to a higher estimation in motivational orientation towards MUDM. Moreover, the indirect effect of attitudes on motivation via TK (path: a1*b1) was also significant with a weak effect (a1*b1 = 0.09, 95% CI [0.043, 0.144]).

Based on the interdisciplinary consideration in the above mediation model, the question arises as to the analysis of the subject-specific characteristics of the three sub-groups: STEM, LLA, and SOCS. To examine the specific influencing factors within individual subject groups, regressive correlations are considered with reference to the subject-specific scales below. Table 3 provides an overview of the respective model with the highest variance explanation.

**Table 3:** Overview of the regression models for the three subject clusters.

Subject cluster	Regression model	Regression coefficients and mediation analysis
*SOCS (n* = *73)*	*MUDM ∼ ALDM + TK + TCK*	*F(3, 69)* = *14.60; p < 0.001* *β*_*ALDM*_ = *0.365, t*_*ALDM*_ = *3.795, p*_*ALDM*_ < *0 .001* *95% CI*_*ALDM*_*[0.152, 0.597]*^a^ *β*_*TK*_ = *0.234, t*_*TK*_ = *2.121, p*_*TK*_ < *0.05* *95% CI*_*TK*_*[0.018, 0.443]*^a^ *β*_*TCK*_ = *0.270, t*_*TCK*_ = *2.461, p*_*TCK*_ < *0.05* *95% CI*_*TCK*_*[0.042, 0.528]*^a^ *β*_TK*TCK_ = *0.134,* *95% CI*_TK*TCK_*[0.023, 0.280]*^a^ *R*^2^ = *0.388* *Adj. R*^*2*^ = *0.362* *VIF*_*ALDM*_ = *1.04; VIF*_*TK*_ = *1.37; VIF*_*TCK*_ = *1.36*
*STEM (n* = *82)*	*MUDM ∼ ALDM + TK + TCK*	*F(3, 78)* = *16.02; p < 0.001* *β*_*ALDM*_ = *0.290, t*_*ALDM*_ = *3.099, p*_*ALDM*_ < *0.01* *95% CI*_*ALDM*_*[0.101, 0.478]*^a^ *β*_*TK*_ = *0.294, t*_*TK*_ = *2.709, p*_*TK*_ < *0.01* *95% CI*_*TK*_*[0.029, 0.529]*^a^ *β*_*TCK*_ = *0.234, t*_*TCK*_ = *2.174, p*_*TCK*_ < *0.05* *95% CI*_*TCK*_*[0.003, 0.476]*^a^ *R*^2^ = *0.381* *Adj. R*^*2*^ = *0.357* *VIF*_*ALDM*_ = *1.10; VIF*_*TK*_ = *1.48; VIF*_*TCK*_ = *1.45*
*LLA (n* = *80)*	*MUDM ∼ ALDM + TK*	*F(2, 77)* = *62.08; p < 0.001* *β*_*ALDM*_ = *0.503, t*_*ALDM*_ = *6.656, p*_*ALDM*_ < *0.001* *95% CI*_*ALDM*_*[0.328, 0.655]*^a^ *β*_*TK*_ = *0.450, t*_*TK*_ = *5.956, p*_*TK*_ < *0.001* *95% CI*_*TK*_*[0.292, 0.613]*^a^ *β*_ALDM*TK_ = *0.161* *95% CI*_ALDM*TK_*[0.052, 0.298]*^a^ *R*^2^ = *0.617* *Adj. R*^*2*^ = *0.607* *VIF*_*ALDM*_ = *1.15; VIF*_*TK*_ = *1.15*

a Confidence intervals were calculated via BCa bootstrapping with 10,000 BCa samples.

Within the three subject clusters (SOCS, STEM, and LLA), TK and ALDM continue to emerge as significant predictors of motivational orientation in each of the three regression models (Table 3). For subject clusters STEM and SOCS, independent variables TK, TCK, and ALDM provide the highest variance clarification with *R*^*2*^_*SOCS*_ = 0.381 and *R*^*2*^_*STEM*_ = 0.388, respectively. For cluster LLA, the variance is explained by *R*^*2*^_*LLA*_ = 0.617 for independent variables TK and ALDM.

**Fig. 4:**
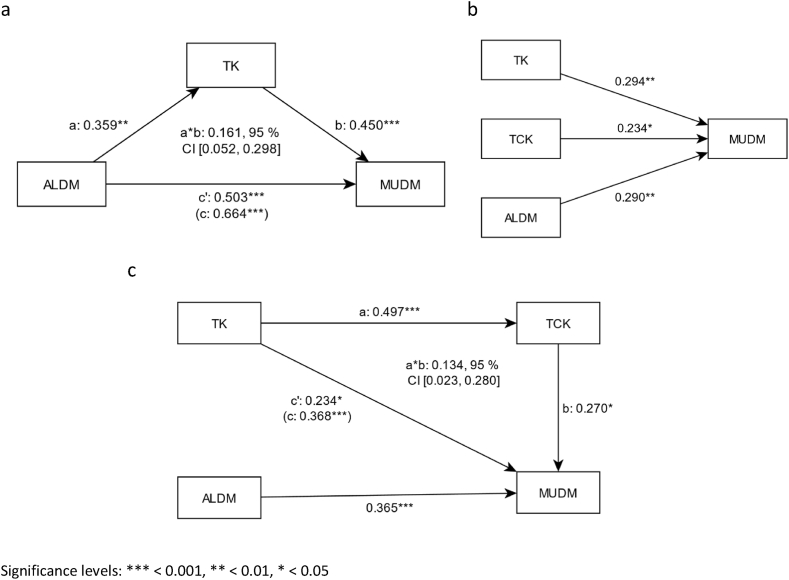
Mediation analyses of subject clusters LLA (a), STEM (b), and SOCS (c).

The additional mediation analyses provide further information about the correlation of the variables within the individual models (Fig. 4). The analysis of the relationship between attitude and motivation is partially mediated in the subject cluster LLA via TK (Fig. 4a). This mediation is rated as weak (a*b = 0.161, 95% CI [0.052, 0.298]). Significantly higher influences on motivation were exerted by the direct paths of ALDM (*β* = 0.503, *p <* 0.001) and *TK* (*β* = 0.450, *p <* 0.001). In subject cluster SOCS (Fig. 4c), the influence of TK on MUDM is partially mediated by TCK (a*b = 0.134, 95% CI [0.023, 0.280]. In addition to the indirect effect, stronger direct influences of variables ALDM (*β* = 0.365, *p <* 0.001), *TK* (*β* = 0.234, *p <* 0.05), and *TCK* (*β* = 0.270, *p <* 0.05) on MUDM are evident. In the STEM cluster, there are no mediation effects (Fig. 4b), but only the direct effects of predictors ALDM (*β* = 0.290, *p <* 0.01), *TK* (*β* = 0.294, *p <* 0.01), and *TCK* (*β* = 0.234, *p* < 0.05) on motivational orientation were observed (Table 3).

## 5 Summary and discussion

The present study aims for a comparative analysis of STEM, LLA, and SOCS student teachers' self-assessments of their digitalisation-related competencies and their attitudes towards using digital media for learning (RQ 1). The second research objective of the study is to analyse the motivational orientation of student teachers towards the use of digital media in the classroom as a function of their self-assessments of the knowledge facets covered and their attitudes towards using digital media for learning (RQ 2).

Summarizing the group comparisons of the first research objective, STEM student teachers rated their technological content knowledge (TCK) and technological knowledge (TK) higher in comparison to both LLA and SOCS student teacher groups. This finding is consistent with the hypothesis. Technological pedagogical knowledge (TPK) is rated statistically significantly higher by STEM teaching students compared to the surveyed LLA teaching students. Not conforming to the hypothesis, the group comparisons do not demonstrate any differences regarding attitudes towards using digital media for learning (ALDM) or motivational orientation towards using digital media in the classroom (MUDM). The study's results show that student teachers have comparable attitudes towards using digital media for learning (ALDM) and comparable motivational orientations towards the use of digital media in the classroom regardless of the subject cluster.

Regarding the second research objective, it should be noted that the motivational orientation towards the use of digital media is influenced across the subjects by the student teachers' self-assessments of their technological knowledge (TK), their technological pedagogical knowledge (TPK), and their attitudes towards using digital media for learning (ALDM). According to this, student teachers who assume that the use of digital media in school promotes self-determined learning, motivates students to learn, or opens up the scope for creativity in learning are more motivationally oriented towards using digital media in the classroom.

When cross-subject modelling the attitudes towards using digital media for learning, technological knowledge (TK) and technological pedagogical knowledge (TPK) are relevant for explaining the motivational orientation towards the use of digital media. However, in the exploratory analysis within individual subject clusters, in addition to attitudes, technological knowledge (TK) and, in the case of STEM and SOCS subgroups, technological content knowledge (TCK) also become relevant for explaining the use of digital media. In the subject-specific analysis, it seems plausible that subject-specific predictor TCK (in the subject-didactic sense) replaces predictor TPK (media pedagogical aspect), which had previously been significant in the cross-subject model.

This study’s results thus confirm existing findings from comparable studies [23,65] and furthermore extend them with respect to the identified disparities between the three subgroups: STEM, LLA, and SOCS teaching students. Thus, it can be stated for the overall sample that in addition to the training of technology-related competencies and media pedagogical competencies, the formation of attitudes during the course of studies appears to be of central importance in the promotion of a positive motivational orientation towards the use of digital media among student teachers. When considering individual subject clusters, it is observable that technological didactic knowledge (TCK) is relevant to the explanation of motivational orientation for STEM and general education student teachers and not for LLA student teachers. The causes of this phenomenon can only be conjectured. Clarifying these facts in an evidence-based manner is a central desideratum for future research.

Due to its explorative approach, the present study cannot provide a conclusive answer as to what is the actual influence of student teachers’ motivational orientation towards the use of digital media in the classroom by subject cluster. When interpreting the study's findings, the fact that the data are cross-sectional and that the limitations of the study can be cited regarding possible intragroup effects in individual subject clusters should be taken into account. Detailed analyses will follow in order to enable further statements about the training of teachers at the level of individual teaching subjects and under the consideration of local higher education framework conditions. As mentioned in other studies (see Section 2), the use of self-assessments is not without problems since findings from self-reports show that the individual's assessments of their knowledge and performance can be subject to bias [75]. Despite the methodological caveats and consideration of research economics, this study provides data-based evidence to further develop higher educational offerings. What are they in detail?

Accordingly, the results of the interdisciplinary analysis do not suggest the need for subject-specific support measures but focus on the training of technological competencies and the promotion of positive attitudes across all subjects. According to the findings, it seems reasonable to additionally promote technological content competencies (TCK) in the context of the individual subject didactics [76]. The quantitative findings from regression models are supported by the interview study conducted with student teachers in a digitalisation-related context by Zinn et al. [7]. The findings of this interview study show that students would like to see more role models and the stronger integration of digital technologies in didactic subject courses and additional technology-related courses with a focus on pedagogical didactic mapping, among other things [77]. If one follows the findings of behavioural research that actions are not only significantly guided by attitudes [20,21] but also that the behaviour of teachers and learners displayed in courses influences (digitalisation-related) attitudes [22], extended (active) options for action in teacher training courses could contribute to promoting a positive motivational orientation towards the use of digital media in teaching.

### 5.1 Funding reference

This article was written as part of the project "MakEd_digital" under the funding code 01JA2026A. This project is part of the “Qualitätsoffensive Lehrerbildung”, a joint initiative of the Federal Government and the *Länder* which aims to improve the quality of teacher training. The programme is funded by the 10.13039/501100002347Federal Ministry of Education and Research. The authors are responsible for the content of this publication.

## Declarations

The authors hereby declare that the study has been approved for conduct by the Ethics Committee of the University of Stuttgart (Az 23–017).

## Author contribution statement

Marcus Brändle, Christina Sotiriadou: Conceived and designed the experiments; Performed the experiments; Analyzed and interpreted the data; Wrote the paper.

Bernd Zinn: Conceived and designed the experiments; Wrote the paper.

## Data availability statement

Data will be made available on request.

## Declaration of competing interest

The authors declare that they have no known competing financial interests or personal relationships that could have appeared to influence the work reported in this paper.

## Footnotes

2 Confidence intervals calculated per BCa bootstrapping with 10000 BCa samples.

## Footnotes

1 According to van Ackeren et al. [1, p. 108], digitisation-related competence includes "media pedagogical competence (in the sense of knowledge, ability, reflexivity and professional ethical attitude, with regard to teaching and to school as an organisation), subject didactic competence (change/expansion of learning objects and subject-specific competencies to be promoted under the conditions of digitisation and digitality) and informatics competence (algorithmic thinking, data literacy, computational thinking and data security)".
