# Supplementary material for: Self-assessments, attitudes, and motivational orientations towards the use of digital media in teaching a comparison between student teachers of different subject clusters
Source: Heliyon. 2023 Aug 29;9(9):e19516. doi: 10.1016/j.heliyon.2023.e19516 (PMC10558735; doi:10.1016/j.heliyon.2023.e19516)
Supplement: Multimedia component 1 [file mmc1.docx]

| **Questionnaire for self-assessment of digitization-related competencies** |
| --- |

**Note: The items and scales were applied in German. The adapted scales have not been piloted in their translated version.**

**Preliminary remarks**

The use of new digital technologies in the classroom and for self-learning phases at home is becoming increasingly commonplace. That's why it's important to be prepared for this already during your studies. With the present survey, we would like to see where the students stand and, if necessary, make improvements in teaching.

In addition to general data, we would like to use this questionnaire to collect your self-assessment of your digitization-related skills. The survey is purely for scientific purposes. All data will be treated strictly confidentially and in accordance with data protection regulations and will be evaluated exclusively anonymously. No personal data will be passed on to third parties. To be able to follow the development during the study, it is necessary to enter a personal code.

By submitting the questionnaire, you consent to the collection and processing of your data for scientific purposes in accordance with the above provisions.

| **Code:** | | | | | | | | |
| --- | --- | --- | --- | --- | --- | --- | --- | --- |
|  | | | | | | | | |
|  |  |  | |  | | First letter of your **mother's first name** | | |
|  | | | | | | | | |
|  |  |  | |  | | Second letter of your **mother's first name** | | |
|  | | | | | | | | |
|  |  |  | |  | | First letter of your **father's first name** | | |
|  | | | | | | | | |
|  |  |  | |  | | Second letter of your **father's first name** | | |
|  | | | | | | | | |
|  |  |  | |  | | **Your birth month** with two digits (e.g. 05 for May) | | |
|  | | | | | | | | |
|  |  |  | |  | | **Your year of birth** with two digits (e.g. 89 for 1989) | | |
|  | | | | | | | | |
|  | | |  | |  | |  |  |

**Demographics:**

| Sex:  female  divers  male |
| --- |
| Age: ___ |
| At which university do you study?  _______________________________________________________________ |
| Study subjects:  Subsidiary subj.  Main subj.   1. Subject ________________________________ 2. Subject ________________________________ |
| How many semesters are you currently in college?  Master  Bachelor  Semester: ___ |
| What education science courses have you already taken? |
| Which didactic courses have you already attended? |
| Did you learn a profession, if yes, which one? |
| Do you have a degree, if so, which degree program? |

Please answer the following questions on a scale from "strongly disagree " to "strongly agree". If you are unsure or neutral about your answer, please select the answer category "neither agree nor disagree".

|  | strongly disagree | disagree | neither agree nor disagree | agree | strongly agree |
| --- | --- | --- | --- | --- | --- |
| **TK (Technology Knowledge) [taken unchanged from 1]** |  |  |  |  |  |
| 1. I know how to solve my own technical problems. |  |  |  |  |  |
| 1. I can learn technology easily. |  |  |  |  |  |
| 1. I keep up with important new technologies. |  |  |  |  |  |
| 1. I frequently play around the technology. |  |  |  |  |  |
| 1. I know about a lot of different technologies. |  |  |  |  |  |
| 1. I have the technical skills I need to use technology. |  |  |  |  |  |
| **CK (Content Knowledge) [adapted from 1]** |  |  |  |  |  |
| 1. I have sufficient subject knowledge in my 1^st^ subject. |  |  |  |  |  |
| 1. I can use a subject-specific way of thinking in the 1^st^ subject. |  |  |  |  |  |
| 1. I have various strategies of further developing my subject-specific knowledge in my 1^st^ subject. . |  |  |  |  |  |
| **PK (Pedagogical Knowledge)[taken unchanged from 1]** |  |  |  |  |  |
| 1. I know how to assess student performance in a classroom. |  |  |  |  |  |
| 1. I can adapt my teaching based-upon what students currently understand or do not understand. |  |  |  |  |  |
| 1. I can adapt my teaching style to different learners. |  |  |  |  |  |
| 1. I can assess student learning in multiple ways. |  |  |  |  |  |
| 1. I can use a wide range of teaching approaches in a classroom setting. |  |  |  |  |  |
| 1. I am familiar with common student understandings and misconceptions. |  |  |  |  |  |
| 1. I know how to organize and maintain classroom management. |  |  |  |  |  |
| **PCK (Pedagogical Content Knowledge) [adapted from 1, 2]** |  |  |  |  |  |
| 1. I can select effective teaching approaches to guide student thinking and learning in my 1^st^ subject. |  |  |  |  |  |
| 1. I can help my students understand the knowledge of my 1^st^ subject in a variety of ways. |  |  |  |  |  |
| 1. I can identify the learning difficulties of my students in my 1^st^ subject. |  |  |  |  |  |
| 1. In class, I can stimulate meaningful discussions about the subject content of my 1^st^ subject . |  |  |  |  |  |
| 1. I can motivate students to deal with real-life problems in my 1^st^ subject. |  |  |  |  |  |
| 1. I can support students to organize themselves in learning the subject knowledge of my 1^st^ subject. |  |  |  |  |  |
| **TCK (Technological Content Knowledge) [adapted from 1, 2]** |  |  |  |  |  |
| 1. I know about digital technologies that I can use to help students better understand and apply the content of my 1^st^ subject. |  |  |  |  |  |
| 1. I can use software designed specifically for teaching my 1^st^ subject (e.g., interactive whiteboard media, glossaries, databases, e-learning). |  |  |  |  |  |
| 1. I have experience using software to help me research or retrieve current subject-specific information for my 1^st^ subject (e.g., Wikipedia, digital lesson manager, textbooks as e-books). |  |  |  |  |  |
| 1. I am able to use appropriate software to illustrate the content of my 1^st^ subject (e.g., animations, simulations, interactive 3D models). |  |  |  |  |  |
| 1. I can use specific software to check the level of knowledge of my students. |  |  |  |  |  |
| **TPK (Technological Pedagogical Knowledge) [taken unchanged from 1]** |  |  |  |  |  |
| 1. I can choose technologies that enhance the teaching approaches for a lesson. |  |  |  |  |  |
| 1. I can choose technologies that enhance students' learning for a lesson |  |  |  |  |  |
| 1. My teacher education program has caused me to think more deeply about how technology could influence the teaching approaches I use in my classroom. . |  |  |  |  |  |
| 1. I am thinking critically about how to use technology in my classroom. . |  |  |  |  |  |
| 1. I can adapt the use of the technologies that I am learning about to different teaching activities. |  |  |  |  |  |
| 1. I can select technologies to use in my classroom that enhance what I teach, how I teach and what students learn. |  |  |  |  |  |
| 1. I can use strategies that combine content, technologies, and teaching approaches that I learned about in my coursework in my classroom. |  |  |  |  |  |
| 1. I can provide leadership in helping others to coordinate the use of content, technologies, and teaching approaches at my school and/or district. |  |  |  |  |  |
| 1. I can choose technologies that enhance the content for a lesson. |  |  |  |  |  |
| **TPACK (Technology Pedagogy and Content Knowledge) [adapted from 1, 2]** |  |  |  |  |  |
| 1. I can combine subject knowledge of my 1^st^ subject, digital technologies, and teaching methods in the classroom in such a way that they support the teaching process efficiently. |  |  |  |  |  |
| 1. I can use digital technologies in my 1^st^ subject classroom in a way that promotes the development of learners' subject knowledge. |  |  |  |  |  |
| 1. I use digital technologies intentionally to stimulate meaningful discussions about the content of my 1^st^ subject and I can guide students to use the appropriate tools for online collaboration (e.g., Moodle, online forums, chat programs). |  |  |  |  |  |
| 1. I can plan classroom activities so that students can construct different representations of the content of my 1^st^ subject using the exact digital media that are most appropriate (e.g., digital mind maps, concept maps, wikis, PowerPoint). |  |  |  |  |  |
| 1. I can use digital media and technology to enable my students to engage in self-organized learning activities related to the subject knowledge of my 1^st^ subject (e.g., email, chat programs, blogs, webquests, learning platforms, forums). |  |  |  |  |  |
| 1. I can prepare questions about the subject knowledge of my 1^st^ subject in such a way that my students can independently use the appropriate digital technology to deal with them. |  |  |  |  |  |

| 1. Ich kann digitale Medien so im Unterricht meines 1. Unterrichtsfachs einsetzen, dass Sie den Aufbau der Kompetenzbereiche „Erkenntnisgewinnung“ und „Bewertung und Kommunikation“ bei den Lernenden fördern. |  |  |  |  |  |
| --- | --- | --- | --- | --- | --- |
| 1. Ich kann Unterrichtseinheiten entwerfen, die auf angemessene Weise die Fachinhalte meines 1. Unterrichtsfachs, digitale Medien und didaktische Unterrichtsmethoden so miteinander verbinden, dass die Schüler*innen eigenständig lernen können. |  |  |  |  |  |

|  | Strongly Disagree | Disagree | Agree | Strongly Agree |
| --- | --- | --- | --- | --- |
| **Attitudes [adapted from 3]** |  |  |  |  |
| 1. Digital media should generally be given a strong weighting in school curricula. |  |  |  |  |
| 1. The use of digital media enables self-determined learning to a high degree. |  |  |  |  |
| 1. By using digital media, students can be better motivated to learn. |  |  |  |  |
| 1. Computers and digital media open up scope for creativity in learning. |  |  |  |  |
| 1. The use of digital media at school ensures that children are well prepared for working life. |  |  |  |  |
| 1. Learning with digital media is an efficient form of learning. |  |  |  |  |
| 1. With digital media I can plan and adapt lessons more appropriately for the target group. |  |  |  |  |
| 1. Digital media allow a higher activation of students. |  |  |  |  |
| **Motivation [adapted from 3]** |  |  |  |  |
| 1. I enjoy thinking about how I can use digital media in the classroom (in the future). |  |  |  |  |
| 1. I also inform myself in my leisure which possibilities there are to integrate digital media in my (future) lessons. |  |  |  |  |
| 1. I like to learn how to use digital media (e.g. programs) for my (future) classes. |  |  |  |  |
| 1. I am willing to invest a little more time in my (future) lesson preparation if I can use digital media in my lessons. |  |  |  |  |
| 1. I am very excited to consider how I can better support the learning of my (future) students using digital media. |  |  |  |  |
| 1. Selecting or creating digital media for teaching is one of the most interesting parts of (future) lesson preparation. |  |  |  |  |

References

[1] D. A. Schmidt, E. Baran, A. D. Thompson, P. Mishra, M. J. Koehler, and T. S. Shin, “Technological Pedagogical Content Knowledge (TPACK),” *Journal of Research on Technology in Education*, vol. 42, no. 2, pp. 123–149, 2009, doi: 10.1080/15391523.2009.10782544.

[2] B. Zinn, M. Brändle, C. Pletz, and S. Schaal, “Wie schätzen Lehramtsstudierende ihre digitalisierungsbezogenen Kompetenzen ein?: Eine hochschul- und fächerübergreifende Studie,” *die Hochschullehre*, pp. 156–171, 2022, doi: 10.3278/HSL2211W.

[3] C. Vogelsang, A. Finger, D. Laumann, and C. Thyssen, “Vorerfahrungen, Einstellungen und motivationale Orientierungen als mögliche Einflussfaktoren auf den Einsatz digitaler Werkzeuge im naturwissenschaftlichen Unterricht,” *ZfDN*, vol. 25, no. 1, pp. 115–129, 2019, doi: 10.1007/s40573-019-00095-6.
